# Use of silver nanowires to determine thresholds for fibre length-dependent pulmonary inflammation and inhibition of macrophage migration *in vitro*

**DOI:** 10.1186/1743-8977-9-47

**Published:** 2012-12-02

**Authors:** Anja Schinwald, Tanya Chernova, Ken Donaldson

**Affiliations:** 1 MRC/University of Edinburgh, Centre for Inflammation Research, Queen’s Medical Research Institute, 47 Little France Crescent, Edinburgh, EH16 4TJ, UK; 2 MRC Toxicology Unit, Hodgkin Building, Lancaster Road, Leicester, LE1 9HN, UK

**Keywords:** Asbestos fibre, Silver nanofibres, Aspiration, Clearance, Migration

## Abstract

**Background:**

The objective of this study was to examine the threshold fibre length for the onset of pulmonary inflammation after aspiration exposure in mice to four different lengths of silver nanowires (AgNW). We further examined the effect of fibre length on macrophage locomotion in an *in vitro* wound healing assay. We hypothesised that exposure to longer fibres causes both increased inflammation and restricted mobility leading to impaired clearance of long fibres from the lower respiratory tract to the mucociliary escalator *in vivo*.

**Methods:**

Nine week old female C57BL/6 strain mice were exposed to AgNW and controls via pharyngeal aspiration. The dose used in this study was equalised to fibre number and based on 50 μg/ mouse for AgNW_14_. To examine macrophage migration *in vitro* a wound healing assay was used. An artificial wound was created in a confluent layer of bone marrow derived macrophages by scraping with a pipette tip and the number of cells migrating into the wound was monitored microscopically. The dose was equalised for fibre number and based on 2.5 μg/cm^2^ for AgNW_14._

**Results:**

Aspiration of AgNW resulted in a length dependent inflammatory response in the lungs with threshold at a fibre length of 14 μm. Shorter fibres including 3, 5 and 10 μm elicited no significant inflammation. Macrophage locomotion was also restricted in a length dependent manner whereby AgNW in the length of ≥5 μm resulted in impaired motility in the wound closure assay.

**Conclusion:**

We demonstrated a 14 μm cut-off length for fibre-induced pulmonary inflammation after aspiration exposure and an *in vitro* threshold for inhibition of macrophage locomotion of 5 μm. We previously reported a threshold length of 5 μm for fibre-induced pleural inflammation. This difference in pulmonary and pleural fibre- induced inflammation may be explained by differences in clearance mechanism of deposited fibres from the airspaces compared to the pleural space. Inhibition of macrophage migration at long fibre lengths could account for their well-documented long term retention in the lungs compared to short fibres. Knowledge of the threshold length for acute pulmonary inflammation contributes to hazard identification of nanofibres.

## Background

The determinants of the pathogenicity of fibrous materials include fibre diameter, length and biopersistence which form the basis of the fibre pathogenicity paradigm (FPP) [1]. Studies on asbestos fibres, synthetic vitreous fibres and nanofibres have shown that they all may pose a significant health hazard when inhaled during their manufacture and/or use [2-5]. These various different fibrous materials possess considerable differences in their pathogenicity in keeping with the FPP [6]. It has been accepted for many years that fibre length plays a crucial role in the development of asbestos related diseases. For example Davis *et al*. performed a number of experiments investigating the pathogenicity of various length and compositions of asbestos fibres via the routes of inhalation and intraperitoneal injection [7,8]. The inhalation studies showed that fibres with a significant proportion (~11%) of fibres > 10 μm caused widespread pulmonary fibrosis and cancer whereas shorter fibres (less than 5 μm) and UICC amosite (intermediate length) caused less fibrosis or carcinogenesis. However, the UICC amosite fibres and long asbestos fibres had similar potency in causing mesothelioma whilst virtually no carcinogenicity was seen with short fibres [8]. This study showed for the first time a difference in the fibre lengths required for the induction of lung diseases and the lengths for peritoneal mesothelioma and by analogy pleural mesothelioma. However, the precise threshold lengths for both lung and peritoneal pathology after fibre exposure were unknown at the time. In a recent study we determined the threshold length for fibre-induced pathogenicity in the pleura [9] as being 5 μm, using distinct length classes of silver nanowires (AgNW). The aim of the current study was to determine the threshold length for fibre-induced lung inflammation by comparing the pulmonary inflammatory response to the same panel of different length classes of AgNW after their deposition in the airspaces of the lungs.

The normal lung clearance mechanisms provide a defence mechanism for removing fibre dose and yet selective retention of longer fibres is well-documented [10,11]. For the key fraction of fibres that deposit beyond the ciliated airways and are slowly cleared, macrophage are the central cells involved in phagocytosing and transporting the fibres to the foot of the mucociliary escalator for clearance. We therefore hypothesised that the uptake of long fibres impairs the ability of macrophages to migrate whilst short fibres do not, providing a mechanism for selective retention of long fibres. We addressed this hypothesis using the samples with different fibre length classes and assessed their effects on macrophage migration in an *in vitro* wound closure assay.

## Results

### Length dependent inflammatory response to AgNW in lung at 24 hour

**Figure 1:**
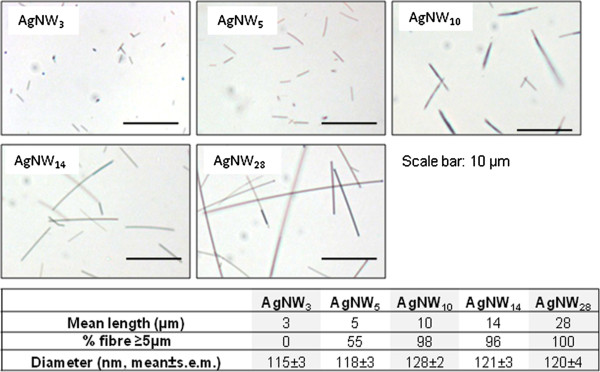
**Characteristics of AgNW panel.** Representative images of the AgNW (1 mg/ml) and their mean length (μm), percentage of fibre ≥ 5 μm and average diameter (nm). Scale bar: 10 μm.

**Figure 2:**
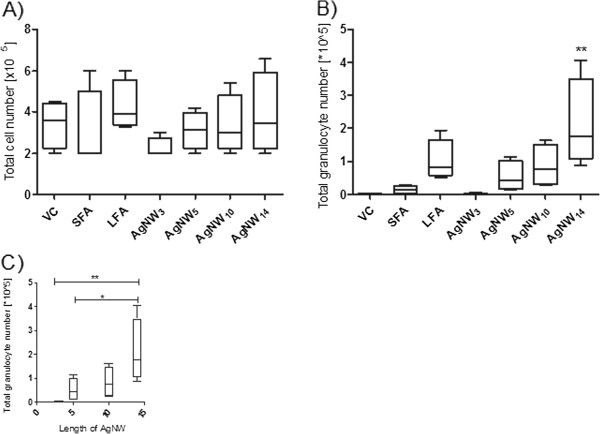
**Length dependent inflammatory response to AgNW in the lungs at 24 hour.** C57/Bl6 mice were exposed to AgNW and control panel by pharyngeal aspiration. At 24 hour post exposure the lungs were lavaged and total cell number (**A**) and total granulocyte number (**B**) were measured. **C**) The acute inflammatory increase was plotted against length of AgNW. Significance indicated compares treatment groups to vehicle control (**B**) and within each treatment (**C**), * indicates p < 0.05, ** indicates p < 0.001. Data represent mean ± SEM of n = 4 mice.

The panel of AgNW used for pharyngeal aspiration is represented in Figure 1. The inflammatory response to the panel of AgNW and control fibres as assessed by the bronchoalveolar lavage fluid (BAL) profile was measured 24 hour post aspiration. An initial dose response series was performed with AgNW_14_ at a dose of 5, 10, 25 and 50 μg/mouse (n = 2) and SFA and LFA at a dose of 10 and 50 μg/mouse (n = 2) (data not shown). The dose of 50 μg/mouse was chosen since it gave a persistent inflammatory response with AgNW_14_ and LFA. The dose was adjusted so that each of the different treatments (Table 1) comprised the similar fibre number. No change in total bronchoalveolar cell number was measured after exposure to equal numbers of the different fibre lengths compared to vehicle control (Figure 2A). However the total number of granulocytes was significantly increased after exposure to AgNW_14_ (Figure 2B). A length dependent trend towards an increase in granulocyte number with AgNW_5_, AgNW_10_ and LFA was observed, although this was not significant (Figure 2B). AgNW_3_ and SFA produced no increase in granulocyte number compared to VC (Figure 2B). The effect of length on inflammation was demonstrated by plotting AgNW length against inflammation and it is clear that the relationship is not linear and that there is a discernible step–increase in granulocyte recruitment at a length between 10 and 14 μm. (Figure 2C). Total amount of protein and increase in membrane permeability by the release of LDH was measured but showed no significant difference between the treatments (data not shown).

**Table 1:** **Calculation for the mass adjustments for equalisation of fibre number*in vivo***

**Particle**	**Calculation to equalise for the same fibre number**	**Dose (μg/mouse)**	**Total fibre number**
**SFA**	Short fibre control	10.7	50.48*10^6^
**AgNW**_**3**_	3/14 × 50	10.7	32.71*10^6^
**AgNW**_**5**_	5/14 × 50	17.9	31.19 *10^6^
**AgNW**_**10**_	10/14 × 50	35.7	28.17 *10^6^
**AgNW**_**14**_	standard	50.0	32.76 *10^6^
LFA*	Long fibre control	50.0	8.24*10^6^

*LFA fibre counting 164,705 fibres/μg on the basis of WHO counting criteria.

### Histological evaluation of lung sections following treatments

**Figure 3:**
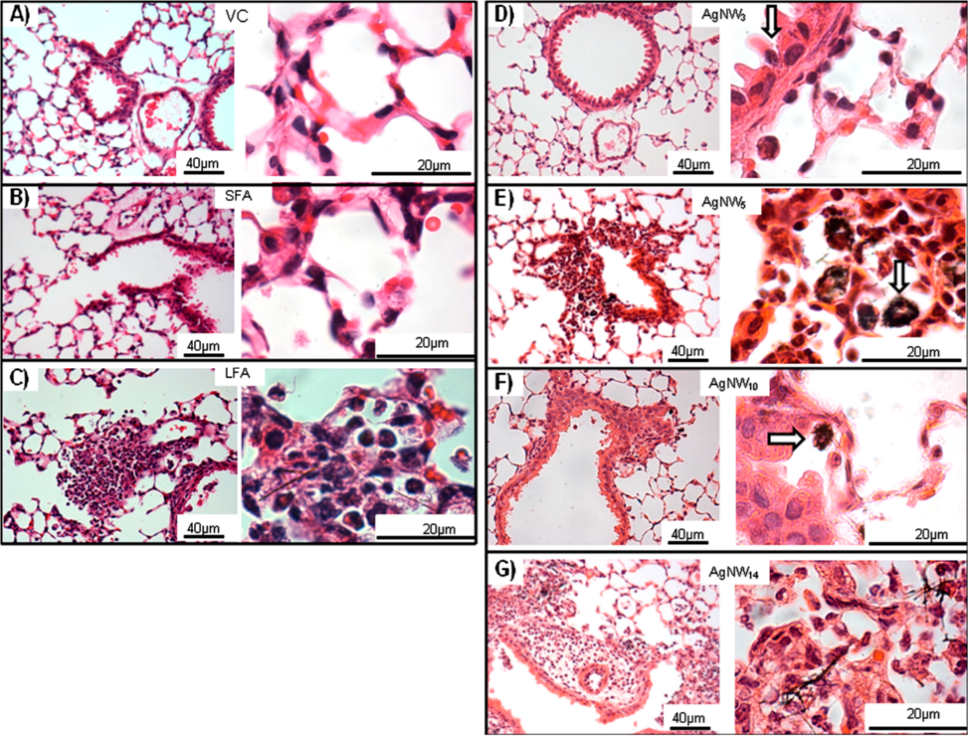
**Lung histology 24 hour post aspiration to AgNW panel and controls.** H&E stained lung sections at lower (left) and higher (right) magnification show normal alveolar structure in the vehicle control (**A**), SFA (**B**) and AgNW_3_ (**D**) sections. Small accumulations of inflammatory cells were seen after AgNW_5_ (**E**) and AgNW_10_ (**F**) exposure whereas a greater amount of inflammatory cell infiltration in the alveoli, peribronchiolar and perivascular regionswere observed after LFA (**C**) and AgNW_14_ (**G**). The white arrows are indicating the areas of fibre accumulation. Abbreviations: al, alveolus; br, bronchiole; v, blood vessel. Representative images are shown, similar results are obtained from 2 mice.

The pathology of the lung exposed to the AgNW panel and controls were examined 24 hour after aspiration. The sections after exposure to VC, SFA and AgNW_3_ showed normal histology of bronchioles, respiratory bronchioles, alveolar ducts and alveoli (Figures 3A, B, D and Additional file 1: Figure S1). AgNW_5_ and AgNW_10_ produced minor granulomas and lymphocyte infiltrates and the majority of the lung histology appeared normal. AgNW_14_ and LFA caused more extensive granuloma and lymphocyte infiltrates which is consistent with the amount of total granulocytes from the lavage fluid (Figure 3E, F, and Additional file 1: Figure S1). Accumulations of nanowires inside alveolar macrophages were common (Figure 3E, F white arrow and Additional file 1: Figure S1). AgNW_14_ produced the strongest response with more granulomatous areas compared to shorter fibres however this was still minor compared to the extensive interstitial thickening and remodelling of the alveolar spaces after LFA treatment (Figure 3C, G, and Additional file 1: Figure S1). Frustrated phagocytosis, classified as incomplete uptake of fibres by cells [12], was observed with AgNW_14_ and LFA.

### Frustrated phagocytosis in alveolar macrophages

**Figure 4:**
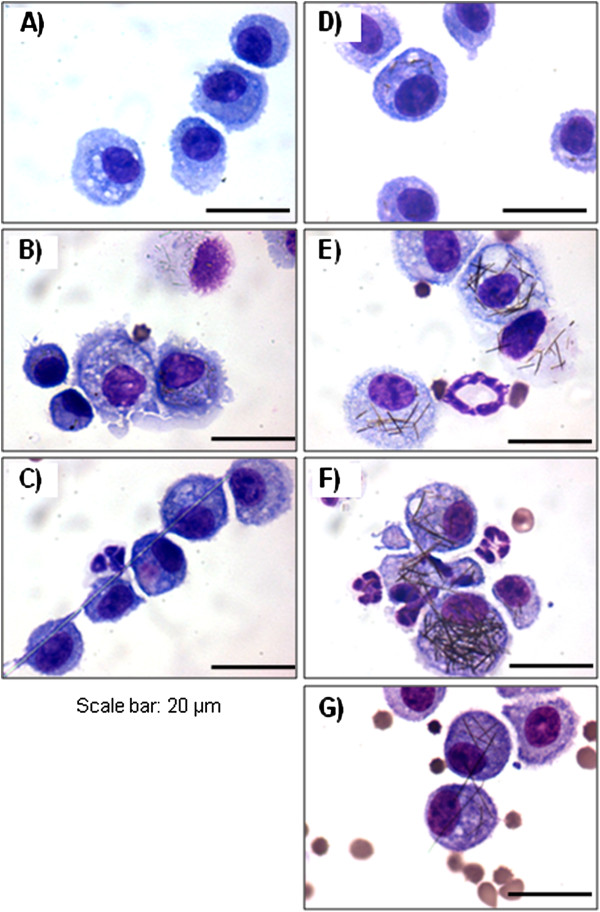
**Uptake of AgNW and controls in alveolar macrophages.** Cytospin images from the lavage fluid 24 hour post aspiration illustrates the complete uptake of shorter fibres including SFA (**B**), AgNW_3_ (**D**), AgNW_5_ (**E**) and AgNW_10_ (**F**) whereas LFA (**C**) and AgNW_14_ (**G**) lead to frustrated phagocytosis in alveolar macrophages. Scale bar 20 μm.

The uptake of different length of AgNW by alveolar macrophages was evident in cytospin images of the broncho-alveolar lavage fluid (Figure 4). Shorter fibres including SFA, AgNW_3_, AgNW_5_ and AgNW_10_ could be completely phagocytosed by alveolar macrophages (Figure 4B, D, E) whereas LFA, and AgNW_14_ were undergoing frustrated phagocytosis indicated by the sharing of fibres between adjacent cells (Figure 4C, G). This is in agreement with frustrated phagocytosis observed in histological sections and the extent of inflammation seen in the lavage fluid showing that frustrated phagocytosis of nanowires correlates with increased inflammation.

### Fibre-length dependent inhibition of locomotion in bone marrow derived macrophages

**Figure 5:**
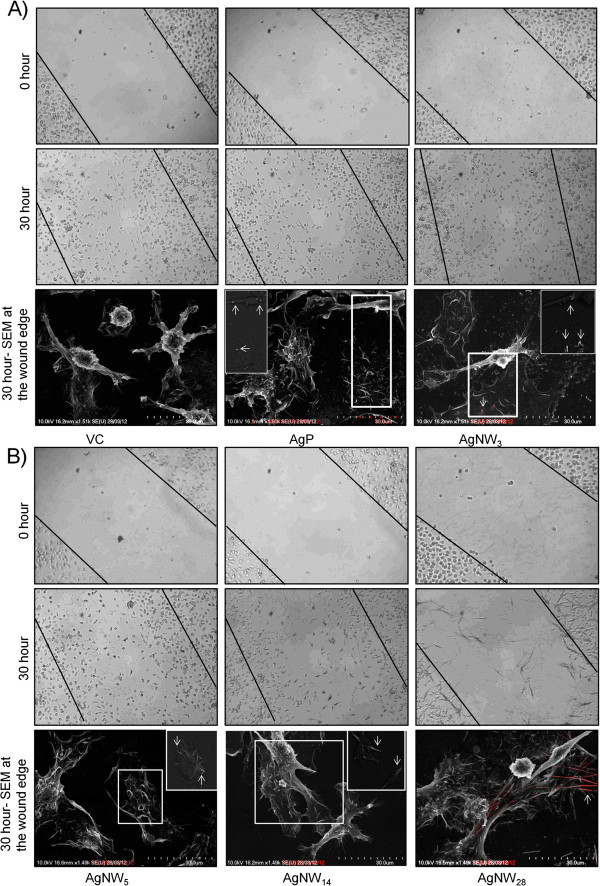
**Fibre-length dependent impaired migration of BMMs in a wound-healing assay.A**,**B**) An artificial wound was created in the BMM monolayer using a pipette tip and the migration of BMMs into the wound was assessed after treatment with VC, AgP, AgNW_3_, AgNW_5_, AgNW_14_, AgNW_28_. The dose was adjusted to fibre number and based on 2.5 μg/cm^2^ for AgNW_14_. Photographs were taken immediately and at 30 hour after creating the wound. For BSE images were taken at the edge of the wound. No impairment of cell adhesion and spreading could be observed and in fact increased cell spreading due to uptake of longer fibres was observed. Nanoparticles and fibres could be visualised underneath the cell membrane as indicated by the white arrow in the inserts which are the non-overlayed BSE images. Representative images are shown; similar results are obtained in 3 independent experiments.

**Figure 6:**
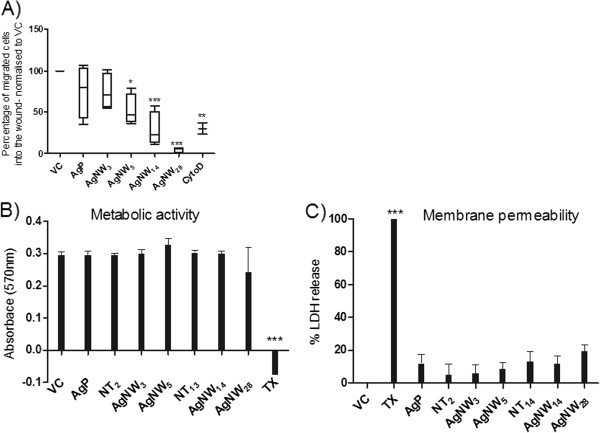
**Inhibition of BMMs migration in a wound-healing assay and cell viability.A**) The number of cell migrated was counted and expressed as percentage of cells migrated into the wound normalised to VC. Significant reduction of migration was seen with AgNW_5_, AgNW_14_, AgNW_28_ and cytochalasin D, a positive control for actin cytoskeleton disruption. **B**) Metabolic activity of BMMs was assessed via the reduction of AlamarBlue® at an absorbance of 570 nm. At the dose used no significant reduction in metabolic activity was measured. **C**) The integrity of the cell membrane after the different treatments was measured via the release of LDH into the cell supernatant and compared to VC and TX, positive control. A sub-lethal dose was chosen at which none of the treatments lead to a significant increase in cell permeability. Significance indicated compares treatment groups to vehicle control, * indicates p < 0.05, ** indicates p < 0.001, *** indicates p < 0.0001 (n = 3). Data represent mean ± SEM of n = 3.

As shown in Figure 5A and Additional file 1: Figure S2, the untreated cells migrated into and repopulated wounds area within 30 hour. Similar wound closure was seen after treatment with silver nanoparticles (AgP) and AgNW_3_ (Figure 5A). A slight decrease in the closure of the wound occurred during treatment with AgNW_5_ whilst BMMs treated with AgNW_14_ and AgNW_28_ (Figure 5B) demonstrate a substantial decrease in the ability to migrate into the wound compared to VC. The number of cells which migrated into the wound were counted and expressed as percentage of migrated cells normalised to VC and this revealed that locomotion was significantly decreased with AgNW_5_, AgNW_14_ and AgNW_28_ (Figure 6A). Cytochalasin D, a positive control which causes impaired migration due to disruption of the actin filaments produced a decrease in locomotion comparable to AgNW_14_ (Figure 6A). Differences in the rate of metabolic activity or cell death could not account for the inability of macrophages to migrate into the wound as the treatment did not interfere with the metabolic activity measured via chemical reduction of Alamar Blue® (Figure 6B) and had no effect on cell membrane integrity measured as the release of lactate dehydrogenase into cell supernatant (Figure 6C). Decrease in cell adhesion has been linked to a decrease in the ability of cell migration. Backscatter scanning electron microscopy (BSE) images confirm that treatment with AgNW did not impair the ability of cell adhesion and spreading (Figure 5A,B). To the contrary, extensive cell spreading is observed in cells treated with long fibres presumably a consequence of BMMs increasing the surface area involved in engulfing the fibres (Figure 5A, B). Using BSE, nanowires and nanoparticles phagocytosed by BMMs could be visualised underneath the membrane as indicated by the arrow (Figure 5A, B).

### Screening for kinase phosphorylation in BMMs after AgNW treatment

**Figure 7:**
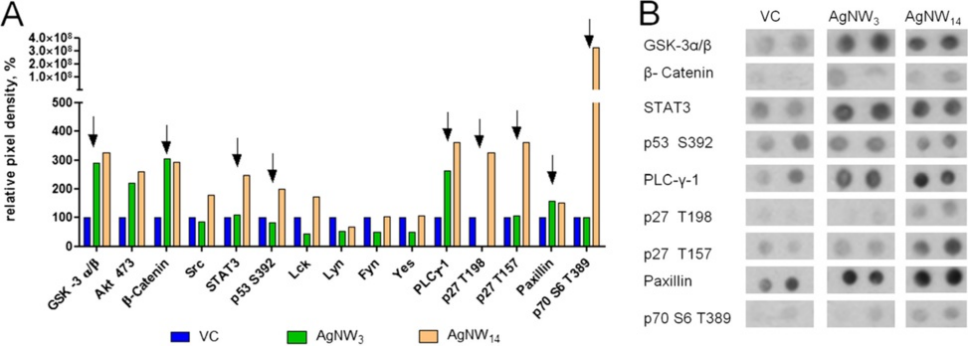
**Phosphoproteomic analysis of BMMs exposed to AgNW.A**) A phospho- kinase array (R&D) was performed to screen the phosphorylation state of 46 kinases in BMMs after treatments with AgNW_3_ and AgNW_14_ whereby 15 kinases showed an increase in phosphorylation relative to VC. **B**) Immunoplot analysis extract of kinases involved in cell migration/mobility and adhesion. Results are based on an initial screening (n = 1).

The phospho- kinase array showed that kinases including GSK-3 α/β, Akt (S473), β-catenin and PLC-γ were activated on treatment with both the AgNW_3_ and the AgNW_14_ which can be explained via a general activation due to phagocytosis of particles (Figure 7A, B). Kinases including STAT3, p53 (S392), p27 (T198), p27 (T157) and p70 S6 (T389) were more activated in AgNW_14_ compared to AgNW_3_ which correlate with the loss of locomotion (Figure 7A, B). Interestingly, the highest level of activation of the cytoplasmic tyrosine kinase Src family members including Src, Lck, Hck and Frg was detected in the AgNW_14_- treated BMM (Figure 7A, Additional file 1: Table S1). A summary of the relative pixel intensities of all kinases can be found in the Additional file 1: Table S1, Figure S3.

## Discussion

The aim of this study was to investigate length-dependent effects of AgNW in the lungs. To assess the role of fibre length in the initiation of inflammation the BAL profile was assessed after aspiration exposure. To assess the effect of length on macrophage clearance and retention of the long fibre dose we used an *in vitro* wound-healing assay. The latter study aimed to shed light onto the effects of fibre length on clearance of fibre-laden alveolar macrophages via migration from the alveolar region of the lung to the mucociliary escalator.

Fibre dimension is a critical factor for lung diseases after inhalation exposure of various forms of fibrous materials as demonstrated in a number of experimental studies, with long fibres being more pathogenic than short ones [7,8,13,14]. We recently reported a length threshold of 5 μm for inflammation in the pleural cavity using the same range of tightly length –defined silver nanowires used here [9].

Long term inhalation studies in rat have been performed with amosite asbestos preparations of short (1% > 5 μm), medium (UICC reference fibre) and long (30% > 5 μm, 11% > 10 μm). These studies showed that the short fibres and the slightly longer UICC sample were low in pathogenicity whilst the long fibres induced extensive fibrosis and pulmonary adenomas. This was in contrast to the studies performed using direct injection of the same samples of amosite asbestos into the peritoneal cavity which showed that UICC amosite was sufficiently long to cause mesothelioma in rats at the same frequency as long amosite fibres [8]. This supports findings of the present study, that the length threshold for fibre effects in the lung is different to the length threshold for fibre effects at the mesothelial surface, the lung threshold being a higher value. More recent studies on adverse effects of nanofibres have shown a positive correlation between increasing fibre length and greater lung and pleural/peritoneal inflammation [4,15,16]. Poland *et al.* showed severe lung and peritoneal inflammation after aspiration exposure and direct intraperitoneal injection in mice of long (24 μm) nickel nanowire but only very mild diffuse alveolitis and mild peritoneal inflammation after exposure to short (4 μm) nickel nanowires [4]. Similar results were obtained from studies on short (<1 μm) and long (>13 μm) carbon nanotubes where the resultant pathophysiological response was compared to short and long amosite asbestos fibres [15-17]. These studies have shown that carbon nanotubes (CNT) induce a length- dependent inflammation in the peritoneal and pleural space of mice [15-17].

We recently investigated the threshold length for pleural inflammation and reported that various forms of high aspect ratio nanomaterials including amosite asbestos fibres, carbon nanotubes, nickel nanowires and the silver nanowires used here, showed a clear length threshold of 5 μm for initiation of an inflammatory response in the pleural space after direct intrapleural injection [9]. The data clearly showed that fibres below 5 μm in length were non-inflammatory and that fibres 5 μm in length and longer caused extensive recruitment of inflammatory cells [9,18] to the pleural space.

The current study addressed the lack of knowledge regarding the threshold length for acute pulmonary inflammation after deposition in the airspaces of the lungs. The pulmonary response to AgNW reported here showed a threshold length for the significant recruitment of inflammatory cells of 14 μm compared to the 5 μm fibre threshold length for pleural inflammation [9,18]. The use of size categories means that the actual threshold could lie anywhere between 11- and 14 μm since the size category below, at which no inflammation was produced was 10 μm. Our finding of a longer threshold in the lungs is consistent with previous reports implicating longer fibres in the development of lung carcinoma compared to mesothelioma [8,9]. The different length thresholds for pulmonary versus pleural inflammation can be explained as a consequence of the different mechanisms of fibre clearance from the lung and the pleural/ space. Clearance of fibres from the pleural space is via lymphatic drainage to mediastinal lymph nodes through stomata, pores in the parietal pleura which are around 0.5–10 μm in diameter [9]. Therefore a size- restricted clearance occurs in the pleural space leading to a retention of fibres which cannot negotiate the stomata and subsequent recruitment of inflammatory cells [15]. The clearance mechanism in the lung relies on alveolar macrophage phagocytosis and migration to the foot of the mucociliary escalator and is discussed in more detail below.

While deposition of AgNW_14_ in the airspaces lead to a significant increase in inflammatory cells in the broncho-alveolar lavage fluid (BAL), shorter fibres including SFA, AgNW_3_, AgNW_5_ and AgNW_10_ only produced a mild, non significant, increase in the total granulocyte number. It was noticeable that the length-dependent response was not linear and showed a marked step-increase in the inflammation between 10 and 14 μm. Histological examination of lung sections showed no recruitment of inflammatory cells after exposure to SFA or AgNW_3_. Minor aggregations of inflammatory cells around terminal bronchioles and in alveolar airspaces were observed with AgNW_5_ and AgNW_10_ but these were more extensive in the AgNW_14_ and LFA samples showing early stages of granuloma development in the terminal airways/ proximal alveolar region. The histological response to LFA was strongest showing extensive aggregates of inflammatory cells. However, the BAL fluid from LFA-exposed mice, whilst showing an increase in the mean number of granulocyte that was almost 4 times the mean number in the mice exposed to SFA did not attain significance. Additionally, lavage from lungs exposed to LFA resulted in a smaller number of BAL granulocytes than AgNW_14_ and this contrasted with the histology data that showed more lung inflammation with LFA than AgNW_14_. The low PMN in the lavage from LFA-exposed lungs was possibly due to a poor return from the inflammation-congested areas of the lungs. The final scoring of LFA equal to AgNW_14_ for BAL granulocytes (Table 2) therefore took into account both the histological and lavage data to provide the best assessment of their relative inflammogenicity. The lack of an inflammatory response with shorter AgNW, which can be considered to be equivalent to silver particles, is consistent with experimental studies demonstrating the minimal lung toxicity or inflammogenicity of nanosilver in a subacute murine and 28-day rat inhalation model [19,20]. The role of silver ion release and its impact on silver nanoparticle toxicity is a controversial topic and experimental situations where soluble silver has been shown to play a role is mainly based on extremely high doses [21]. There is no reason to believe that silver ions played any role here as discussed extensively in our recent paper using the same panel [9] due to the low toxicity of silver nanoparticles and their proclivity to form silver chloride in biological systems. The study presented here focuses on short-time response due to the fact that the AgNW used in this study appear not to be biopersistent beyond a few days [9]. It is highly likely that the same length dependent effects would be shown by wholly biopersistent fibres and that the inflammation would be chronic. However, the determination of the threshold length for long term chronic inhalation studies is of utmost importance and biopersistence remains a key factor in the pathogenicity of long nanofibres.

**Table 2:** Summary of the length-dependent effects of fibres in the lungs

		**Endpoint**				
		BAL Granulocytes^*^	Lung Histology^**^	Frustrated phagocytosis^***^	Impaired migration^****^	Δ kinase phosphorylation^*****^
**Treatment**	**Control**	+	-	No	-	NA
	**SFA**	+	-	No	NA	NA
	**LFA**	+++	++++	Yes	NA	NA
	**AgNW**_**3**_	+	-	No	-	+
	**AgNW**_**5**_	++	+	No	+	+
	**AgNW**_**10**_	++	+	No	++	NA
	**AgNW**_**14**_	+++	+++	Yes	+++	++

* BAL granulocytes: <0.5 million (+); 0.5-1.0 million (++); 1–2 million (+++).

** Lung histology subjectively scored on the basis of the severity of lung inflammation (see results).

*** Frustrated phagocytosis on the basis of evaluation of cytospins for presence of frustrated phagocytosis.

**** Impaired migration on the basis of degree of significant impairment compared to control (see Figure 6A).

***** Δ kinase phosphorylation based on subjective examination the levels of phosphorylation of the treated compared to the control levels of phosphorylation (see Figure 7). *NA* = not applicable.

The clearance efficiency of deposited fibres from the lower respiratory tract plays a major role in the development of pulmonary diseases since the retained dose, which is the dose accumulated in the alveolar region of the lungs after clearance mechanism accounts for the chronic pathogenic effects of inhaled fibres [22].

Fibres deposited in the conducting airways are cleared rapidly via cilia in the mucociliary escalator and subsequently swallowed or expectorated. If fibres reach the respiratory bronchioles, alveolar ducts or alveolar sacs they are cleared slowly via alveolar macrophages (AM) phagocytosing the deposited fibres and transporting them upwards to the ciliated airways for mucociliary clearance. It has been noted in many studies that there is selective retention of long biopersistent fibres of various sorts from the slow-clearing compartment with more effective clearance of the shorter fibres [11,23].

The mechanism by which fibre-laden alveolar macrophages are drawn to the terminal bronchioles at the foot of the ciliated airways is obscure. Possible explanations include the passive transport with alveolar fluid or amoeboid movement of AM either by random migration or directed migration along a chemotactic gradient [24]. Other pathways of clearance, usually only important during high dust exposure or disease are intra- and transcellular pathways by which fibres can reach lung interstitium and lymph nodes from where they subsequently may reach the blood stream [22,25,26]. A recent study investigated the effects of MWCNT on cell migration and adhesion of human dermal fibroblasts and murine fibroblasts, reporting a significant decrease in cell adhesion which was confirmed by the decrease of mRNA levels of important cell adhesion proteins FAK, fibronectin and laminin [27]. In addition fibroblast migration in a wound- healing assay was greatly impaired by MWCNT treatment and was accompanied by cytoskeletal derangement [27], although it should be noted that the dose of MWCNT used in this study was cytotoxic to human fibroblast which could influence the adherence and migration properties of the cells.

We set out to investigate whether fibre length has an effect on the migration behaviour of BMMs *in vitro* after fibre exposure using a wound-healing assay, a surrogate for *in vivo* clearance of fibrous material to the mucociliary escalator by AM migration. We used a very low dose to study effects on BMM migration, based on 2.5 μg/cm^2^ for AgNW_14_ and all the other length classes were adjusted to provide the same fibre number. The dose used had no significant effect on cell metabolism or cell viability for any of the length classes, ensuring that the observed effects were not due to simply impaired cell viability. A clear length-dependent trend for inhibition of BMM cell migration was measured at fibre lengths of 5, 14 and 28 μm and at 28 μm there was more-or less complete inhibition of motility. Presumably as a consequence of cell surface extension of AM during engulfment of long fibres, increased cell spreading was observed and correlated with increased inhibition of migration. The BMMs in this study had an average diameter of 13 μm [28] whilst human alveolar macrophages have an average diameter of 21 μm [29]. Due to this difference the threshold length for clearance of fibres by alveolar macrophages in humans may be slightly higher.

An initial screening of 46 kinase phosphorylation sites was performed to get a snapshot of the kinase activation status of fibre- treated BMMs. We hypothesised that this might reveal whether there was any fundamental change in the activation/metabolic state of the cells when they were impaired in their ability to migrate by phagocytosing long fibres. The 3 μm long exposed cells act as a control for normal phagocytosis and when compared to the untreated kinase profile this showed most of the kinases (~32) remained unchanged. The kinases GSK-3 α/β, Akt 473, β-catenin and PLC-γ showed activation in all treatment groups suggesting a link to normal phagocytosis. Comparing the kinase phosphorylation profile of BMM exposed to 3 μm with those exposed to 14 μm should reveal differences associated with the long fibre-dependent loss of motility and 5 kinases out of 46 (STAT3, p53 (S392), p27 (T198), p27 (T157) and p70 S6 (T389)) exhibited increased phosporylation with AgNW_14_ compared to AgNW_3_. Tyrosine kinases of the Src family were noticeably induced by treatment with long fibres. These kinases have been implicated in intracellular signalling in macrophages influencing the amplitude of many pathways [30]. One of the Src downstream effectors is STAT3, a major modulator of inflammation, which is required for activation of macrophages [31]. A marked Induction of STAT3 following the AgNW_14_ treatment is a likely result of Src activation. It’s worth mentioning that a constitutive activation of STAT3 is a common feature in many solid tumours [32], therefore persisting activation of STAT3 in a chronic inflammation caused by long fibres may contribute to pro-oncogenic changes. Macrophage motility is known to be negatively regulated by p53 [33] and the latter was induced in BMM treated with longer fibres, and might have contributed to impairment of their migration. The role of p27 in regulation of cellular migration remains unclear, however, our finding would support the reports showing suppression of migration by induction of p27 [34]. No kinases were down-regulated in the long fibre treated BMM compared to the controls confirming that there was no generalised loss of viability associated with failure to migrate. It is reported in the literature that a number of different kinase pathways are involved in the migration and adhesion processes among these are kinases that showed an increase in their phosphorylation state after long fibre treatment [35-41]. The results from the screening of the phosphorylation sites was mainly performed to see if crucial cellular signalling processes are impaired by the treatment of AgNW which might indicate that decreased cellular function, apoptosis/necrosis underlay the decrease in cell mobility. Our results showed that the long-fibre treatment did not negatively affect the cellular function of the BMMs as assessed by metabolic function, loss of membrane integrity or kinase profile. In fact long fibre treatment and inhibition of motility were associated with increased phosphorylation of some kinases involved in migration and adhesion. Clearly the interplay between adhesion and motility is complex since focal adhesion is required for motility and a more sophisticated analysis of mechanism underlying loss of motility with long fiber treatment is required but is outwith the scope of the present paper. The results give an indication that cellular processes are intact in long fiber treated BMM and therefore that inhibition of locomotion can be best explained via a mechanical obstruction to motility. By this we suggest that the presence of long fibres inside the cells physically interferes with the necessary rearrangement of the cytoskeleton, membrane and other structures that are necessary for locomotion. This however has to be confirmed by an in depth investigation of the molecular mechanism involved in inhibition of migration which is beyond the scope of the present manuscript.

## Conclusion

In conclusion we have shown that there are length-dependent effects on the lung and on BMM summarised in Table 2. These show length-dependent increases in inflammation by BAL and severity of lung injury by histology in vivo and evidence of accompanying impairment of macrophage migration by BMM in vitro; frustrated phagocytosis was only evident at the longest fibre lengths indicating that complete uptake of longer fibres still causes cell impairment, in the absence of classical frustrated phagocytosis as we previously reported [9,12]. A threshold length for acute pulmonary inflammation after pharyngeal aspiration of AgNW was evident between 10 and 14 μm in length. This compares with our previous studies on the threshold length for pleural inflammation of 5 μm, determined using a panel that utilised the AgNW used here [9]. No previous study has used tight length- restricted fibre populations to demonstrate thresholds as shown here and in our previous study in the pleural space [9]. The present study showing that the threshold length for induction of pulmonary inflammation is longer than the threshold length for pleura inflammation is in accordance with extrapolations using asbestos fibres [8]. The difference in thresholds can be explained by the differences in clearance mechanism between the lung the pleural space. In the pleural space clearance is through stomata [6] whilst clearance of deposited fibres from beyond the ciliated airways is via uptake by AM and subsequent migration to the mucociliary escalator. Using an *in vitro* wound healing assay we showed that fibre length-dependent macrophage mobility, with a threshold for impairment at a length of 5 μm and increasing impairment with increasing length, until at 28 μm there was almost complete inhibition of motility. An explanation for the decrease in locomotion could be mechanical obstruction caused simply by the bulky long fibres interfering with the movement process since there was no loss of viability or respiration in the long fibre-treated cells and an initial screen of a number of 46 kinases showed no decrease kinase phosphorylations. We note however that this is a small-scale aspiration study with acute inflammation and short term inhibition of migration *in vitro* as the endpoints. Our results need to be confirmed in long term inhalation studies using a range of different nanofibres at plausible exposure before we can confidently utilize these thresholds for risk assessment and in benign-by-design for nanofibres.

## Methods

### Particle panel

The panel of particles investigated here consisted of four silver nanowires (AgNW) samples (Figure 1), a silver nanoparticle control (AgP) and two amosite asbestos samples, long amosite asbestos (LFA) and short amosite asbestos (SFA) as previously described in Schinwald *et al*. [9]. AgP was purchased from Nanostructured & Amorphous Materials, Inc. with a diameter of 35 nm, a purity of 99,5% and a specific surface area of 30–50 m^2^/g. The AgNW samples were kindly provided by Seashell Technology, San Diego (http://www.seashelltech.com). The polyol process was used for the synthesis of the AgNW which is described in patent number 7,922,787 B2. Detailed description of particle panel characteristics including concentration of soluble metal and dissolution can be found in Schinwald *et al*. [9]. AgNW synthesis and reaction conditions to obtain different lengths did not affect the chemical composition of the different nanowires and no coating of the nanowires was performed. The panel was dispersed in isopropanol and diluted to a working concentration of 1 mg/ml in 0.5% bovine serum albumin (BSA; Sigma-Aldrich, Poole, UK)/saline. For light microscope images the AgNW (1 mg/ml) were were mixed with 10 μl of glycerol (Sigma-Aldrich, Poole, UK) to reduce the flow of AgNW. The suspension was placed on glass slide and covered with a glass coverslip and sealed [15]. Images were captured at x40 magnification using QCapture Pro software (Media Cybernetics). As a control panel mixed length amosite asbestos enriched for long fibres (100% fibres ≥5 μm, 50.3% fibres >15 μm, 35.2% fibres >20 μm), hereafter referred to as long fibre asbestos (LFA), and shortened amosite asbestos (SFA; 3.1% fibres ≥5 μm, mean length 1 μm, mean diameter 300 nm) [42] were used.

### *In vivo*

#### Experimental animals

Nine week old female C57BL/6 strain mice (Harlan, UK) were used in this study. Mice were kept in a group size of five in standard caging with sawdust bedding within a pathogen-free Home Office approved facility. Mice were maintained on a normal 12 hour light and dark cycle. Prior to the treatment mice were kept for 7 days in the facility to acclimatise. The work was carried out by staff holding a valid UK Home Office personal licence under a Home Office approved project licence.

#### Pharyngeal aspiration and bronchoalveolar lavage

 The dose of AgNW panel for pharyngeal aspiration was equalised to fibre number since fibre exposure is regulated on the basis of the fibre number and so relative potency needs to be determined on a per-fibre basis. To equalise for fibre number a dose of 50 μg/mouse for AgNW_14_ was chosen as the standard *in vivo* dose based on previous measurement of membrane integrity and proliferation. Based on 50 μg/mouse for AgNW_14_, concentrations for the other length classes AgNW panel were calculated assuming that fibres thickness was constant in the different length classes (Table 1). Particle panel was dispersed in 0.5% bovine serum albumin (BSA; Sigma-Aldrich, Poole, U.K.)/saline and briefly vortexed to assist dispersion. Vehicle control (VC) consisted of 0.5% BSA/saline.

Mice were anesthetized with isoflurane (2-chloro-2-(difluoromethoxy)-1,1,1 trifluoroethane) and the tongue was gently held in full extension while 50 μl of particle suspension was pipette onto the base of the tongue [43]. The tongue was held extended until at least two breaths were complete. To stimulate inhalation and to induce a gasp reflex the nasal cavities of the mice were covered. Mice were observed until full recovery.

Mice were sacrificed at 24 hour by terminal anaesthesia. 0.5 ml of pentobarbitone (200 mg/ml) (2, 2, 2-Tribromomethanol) was injected in the peritoneal cavity followed by exsanguinations via the abdominal aorta. The thoracic cavity was exposed and the trachea cannulated using a 23 gauge needle and legated. The lungs were lavaged three times with 800 μl of ice-cold sterile saline. The first lavage was retained separately for LDH and total protein measurements and the subsequent lavages were pooled.

Calculation for total fibre number: e.g. AgNW_3_

Length of NW (average) [μm] =3.00

Diameter of NW [μm] =0.115

Density of NW [μg/ml] =1.05 x 10^−7^

(1) $$\begin{array}{rl} \;\text{Volume}\;\text{of}\;\text{NW}\;\left[\text{ml}\right] & \;=3*\prod *{\left(\frac{0.115}{2}\right)}^{2}\\ & \;=0.03116\times 10^{-14} \end{array}$$

(2) $$\begin{array}{rl} \;\text{Weight of NW}\;\left[\text{μg}\right] & =3.116\times 10^{-14}\times 1.05\times 10^{-7}\\ & =3.272\times 10^{-7} \end{array}$$

Dose [μg] per mouse = 10.70

(3) $$\begin{array}{rl} \;\text{Number of NW} & =\frac{10.70}{3.272\times 10^{-7}}\\ & =32719592.52≅32.71\times 106\;fibers \end{array}$$

#### Lung dissection

For histological examination of lung pathology no forgoing BAL was performed and n = 2 for each treatment was used. 10% formalin was instilled into the lungs to fix the lung tissue and removed ‘en block’ with the heart. The lung and heart were kept for 4 hours in fixative prior to processing. The heart was removed and the lung was dissected into individual lobes and placed flat in a tissue cassette. The tissue was dehydrated through graded alcohol (ethanol) and embedded in paraffin. 4 μm sections were cut from the block covering all lobes of the lung and stained with H&E to show gross pathology.

#### Differential cell count/ total protein and lactate dehydrogenase measurement

The cellular fraction was separated from the supernatant of lavage fluid from BAL by centrifugation for 5 minutes at 2000 g at 4°C in a Mistral 3000i centrifuge (Thermo Fisher Scientific, Inc., MA, USA). Total cell count was performed using a NucleoCounter (ChemoMetec, 7 A/S, Allerød, Denmark) and cyto-centrifugation with following Diff-Quik staining using Diff-Quik stainset (Dade Behring Gmbh, Marburg, Germany) were prepared for differential cell counts.

In the supernatant, membrane integrity using the Cytotoxicity Detection Lactate Dehydrogenase kit (Roche 25 Diagnostics Ltd., Burgess Hill, UK) and protein content using the bicinchoninic acid (BCA) protein assay (Sigma-Aldrich, Poole, UK) were measured following the manufacturer’s instructions.

### *In vitro*

#### Generation of bone marrow derived macrophages

Bone marrow-derived macrophages (BMMs) were generated from 8 week old wild-type C57/Bl6 mouse femurs and tibias. In brief, the bone marrow was flushed with PBS using a 24 gauge needle, resuspended, passed through a cell strainer and centrifuged at 15000 rpm for 3 min. Cells were resuspended in red blood cell lysis buffer Hybrid Max™ (Sigma- Aldrich, UK) for 5 min at room temperature. Cells were centrifuged, resuspended in DMEM (Dulbecco's Modified Eagle Medium, Life Technologies) containing 10% FCS, 1% penicillin/ streptomycin and 20% L929 cell media and plated in a 10-cm^2^ non tissue coated dish and cultured for 7 days.

#### Wound healing assay

After 7 day differentiation BMMs were seeded in 24 well plates at a density of 0.5x10^6^/ml in DMEM containing 10% FCS, 1% penicillin/ streptomycin and 20% L929 cell media and culture for 2 days as a confluent monolayer. The medium was replaced with DMEM containing 0% FCS and 1% penicillin/ streptomycin. Cells were treated with AgNW equalised to fibre number. To equalise for fibre number a dose of 2.5 μg/cm^2^ for AgNW_14_ was chosen as the standard *in vitro* dose based on previous measurement of membrane integrity and proliferation. Based on 2.5 μg/cm^2^ for AgNW_14_, concentrations for the other length classes AgNW panel were calculated assuming that fibres thickness was constant in the different length classes (Table 3). For the control particle, AgP, a dose of 0.5 μg/cm^2^ was used, equal to the shortest fibre (AgNW_3_). The panel was dispersed in 0.5% BSA/ cell culture medium (DMEM, 0% FCS) and added to the cells. An artificial wound was created by scraping with a pipette tip and the number of cells migrating into the wound was monitored microscopically and photographs were taken immediately and after 30 hour. Images were acquired through a Zeiss Axiovert S100 microscope equipped with a 30x objective lens and were captured using RS Photometrics CoolSnap. The number of cells migrated into the wound was counted and expressed as percentage compared to vehicle control. Results are given as the mean ± SEM of 3 independent experiments.

**Table 3:** **Calculation for the mass adjustments for equalisation of fibre number*in vitro***

**Length class [μm]**	**Calculation to equalise for the same fibre number**	**Dose (μg/cm**^**2**^**)**	**Total fibre number**
**3**	3/14 × 2.5	0.5	1528953
**5**	5/14 × 2.5	0.9	1568374
**14**	standard	2.5	1638164
**28**	28/14 × 2.5	5	1638164

#### Backscatter scanning electron microscopy

Backscatter scanning electron microscopy is based on elastic scattering of high energy electron further inside the sample. Elements with high atomic numbers (Z) such as silver give a stronger signal then lower Z elements. The signal is in general weak and can therefore only provide a contrast between regions with a larger difference in atomic number [12].

BMM cells were prepared as described above and seeded into 24 well plates on ThermanoxR Plastic Coverslips (NUNC™, Rochester, NY USA) at a density of 0.5*10^6^/ml. The cells were treated for 30 hours using concentration as described above at 37°C in 5% CO2 atmosphere. After the treatment they were washed 5x with 0.1 M sodium cacodylate (pH 7.2) buffer. Cells were fixed overnight in 3% glutaraldehyde/ 0.1 M sodium cacodylate (pH 7.2) buffer and subsequently washed three times in sodium cacodylate buffer.

BSE of carbon-coated specimens was carried out using a Hitachi 4700 II field emission SEM (Hitachi High-Tech, Maidenhead, UK) at a beam accelerating voltage of 10 kV and a working distance of about 8 mm. Secondary electron (SE) and BSE images were taken simultaneously using an annular YAG crystal BSE detector and the upper SE detector to produce perfectly-synchronised image pairs. Both images were superimposed using Adobe Photoshop. The SE and BSE image were converted to grayscale, the BSE image was pasted into the SE image by using the layer function “lighten”. This newly merged image and the SE image were converted to RGB mode, and overlayed by pasting the red channel of the BSE image into the red channel of the greyscale SE image, thus colour coding in red the strong BSE signal from the nanowires, the SE image appearing in grey.

#### Measurement of membrane integrity and proliferation of BMMs

Supernatant of the wound healing assay was collected and analysed for membrane integrity using using the Cytotoxicity Detection Lactate Dehydrogenase kit (Roche Diagnostics Ltd., Burgess Hill, UK) following the manufacturer’s instructions. TritonX (Sigma) was used as a positive control for cell death and was added at a final concentration of 0.1% for 30mins. The supernatant was centrifuged for 5 mins at 2000 rpm, transferred and centrifuged again for 5 mins at 13000 rpm. The conversion of lactate to pyruvate was detected using a microplate reader (BioTek® SynergyHT) to measure the optical density at 490 nm. Results are given as the mean ± SEM of 3 independent experiments.

Cells in the culture dish were used to measure their proliferation and metabolic activity via a chemical reduction of AlamarBlue® (Invitrogen). 150 μl of PBS and 15 μl of AlamarBlue® was added to each well and incubated for 3 hours at 37°C in 5% CO2 atmosphere. Absorbance was monitored at 570 nm and 600 nm as a reference wavelength. Data are normalized to 600 nm value. Results are given as the mean ± SEM of 3 independent experiments.

#### Proteome profiler array

BMMs were differentiated as described above and seeded at 0.5x10^6^/ml in DMEM containing 10% FCS, 1% penicillin/ streptomycin and 20% L929 cell media and culture for 2 days as a confluent monolayer in a 60 mm tissue culture dish. The medium was replaced with DMEM containing 0% FCS and 1% penicillin/streptomycin and the particle panel was added at the concentrations described above and treated for 30 h. The cells were rinsed with cold phosphate-buffered saline (PBS) and immediately solubilised in lysis buffer by pippeting up and down and rocking the cell lysate at 4°C for 30 min. The lysate was centrifuged at 14,000 x g for 5 min, the supernatant was transferred into new test tubes and the protein concentration was measured using the bicinchoninic acid (BCA) protein assay (Sigma-Aldrich, Poole, UK) following the manufacturer’s instructions. 500 μg of lysates were diluted and incubated with the Human- Phospho – MAPK Array Kit (Proteome Profiler™, R&D Systems) as per manufacturer’s instructions. Plots were developed on X-ray films following exposure to chemiluminescent reagents.

#### Statistical analysis

All data are shown as the mean ± s.e.m. and these were analysed using one-way analysis of variance (ANOVA). Multiple comparison were analysed using Tukey-HSD method and in all cases, values of P < 0.05 were consider significant. (GraphPad InStat Software Inc., CA, USA).

## Abbreviations

BSE: Backscatter scanning electron microscopy; BMM: Bone marrow derived macrophages; FPP: Fibre pathogenicity paradigm; LDH: Lactate deydrogenase; LFA: Long fibre amosite asbestos; SEM: Scanning electron microscopy; SFA: Short fibre amosite asbestos; AgNW: Silver nanowire; AgP: Silver nanoparticle; VC: Vehicle control.

## Competing interests

The authors declare that they have no competing interests.

## Authors’ contributions

AS conceived and designed the experiments, analysed the data and wrote the manuscript. KD initiated the study, oversaw all experimental work and contributed to manuscript preparation. TC performed the proteome kinase array and contributed to the manuscript preparation. All authors read and approved the final manuscript.

## Supplementary Material

Additional file 1 **Figure S1.** 24 hour wound healing assay in BMMs. SEM images showing the closer of the wound after 24 hour at different treatments with AgNW. **Table S1:** Phosphoproteomic analysis of BMMs exposed to AgNW. A phospho- kinase array (R&D) was performed to screen the phosphorylation state of 46 kinases in BMMs after treatments with AgNW. Relative pixel density in % compared to VC is shown for all kinases measured (n=1). **Figure S2:** Phospho-kinase array immunoplots. A) VC, B) AgNW3, C)AgNW5, D) AgNW14. Click here for file
