# Supplementary material for: Use of silver nanowires to determine thresholds for fibre length-dependent pulmonary inflammation and inhibition of macrophage migration in vitro
Source: Part Fibre Toxicol. 2012 Dec 2;9:47. doi: 10.1186/1743-8977-9-47 (PMC3546062; doi:10.1186/1743-8977-9-47)
Supplement: Additional file 1 — Figure S1. 24 hour wound healing assay in BMMs. SEM images showing the closer of the wound after 24 hour at different treatments with AgNW. Table S1: Phosphoproteomic analysis of BMMs exposed to AgNW. A phospho- kinase array (R&D) was performed to screen the phosphorylation state of 46 kinases in BMMs after treatments with AgNW. Relative pixel density in % compared to VC is shown for all kinases measured (n=1). Figure S2: Phospho-kinase array immunoplots. A) VC, B) AgNW3, C)AgNW5, D) AgNW14. [file 1743-8977-9-47-S1.pdf]

Use of silver nanowires to determine thresholds for fibre length-dependent pulmonary inflammation and inhibition of macrophage migration *in vitro*

Supplementary Material:

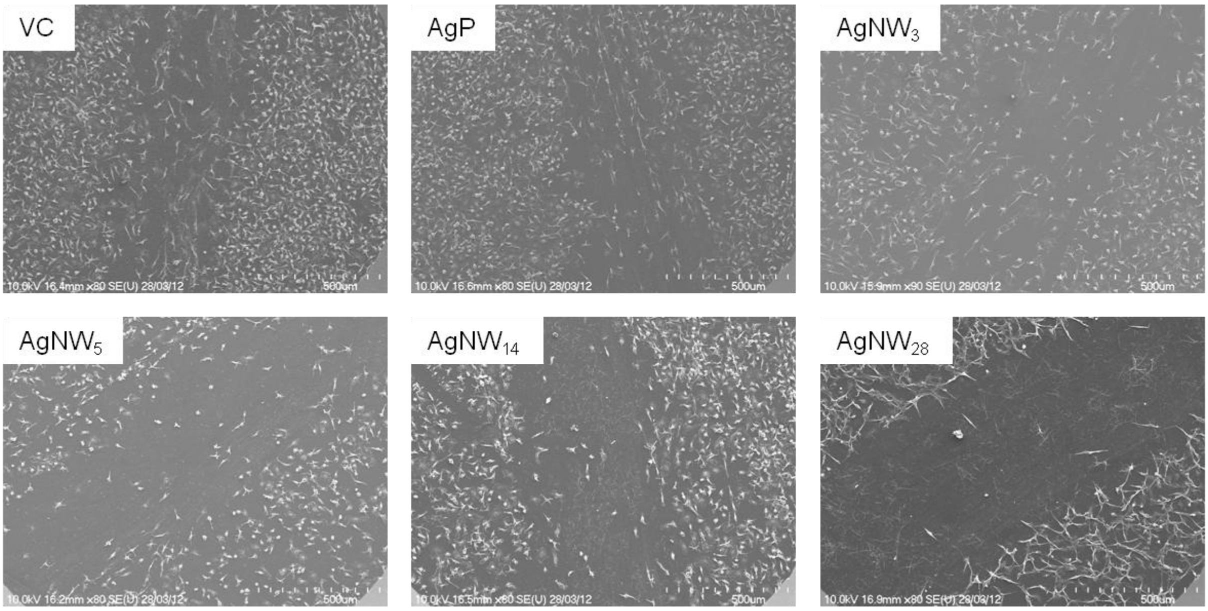

Figure S1: 24 hour wound healing assay in BMMs. SEM images showing the closer of the wound after 24 hour at different treatments with AgNW.

Table S1: Phosphoproteomic analysis of BMMs exposed to AgNW. A phospho- kinase array (R&D) was performed to screen the phosphorylation state of 46 kinases in BMMs after treatments with AgNW. Relative pixel density in % compared to VC is shown for all kinases measured (n=1).

	VC	$AgNW_3$	$AgNW_5$	$AgNW_{14}$
**p38 a**	100	74.85336	159.0873	121.9645
**ERK 1/2**	100	93.41407	86.15658	74.33238
**JNK pan**	100	107.5308	82.98289	86.15175
**GSK -3 a/b**	100	288.9975	256.3521	327.8246
**MEK1/2**	100	115.8622	97.57284	129.8732
**MSK 1/2**	100	77.76693	96.80675	79.47691
**Akt 473**	100	221.5099	137.8032	261.4832
**mTOR**	100	94.12129	92.45151	138.5429
**CREB**	100	52.63563	29.98924	78.0085
**HPS27**	100	128.6365	131.5996	141.1714
**AMPK a2**	100	175.9596	105.3152	140.0264
**β-Catenin**	100	306.2233	249.1231	293.5133
**Chk-2**	100	54.88571	97.62734	103.0031
**Src**	100	86.62845	87.61551	178.408
**Lyn**	100	52.63225	45.04333	67.13648
**Lck**	100	43.72153	32.89619	171.7983
**Fyn**	100	51.26141	57.08296	104.4002
**Yes**	100	50.55822	72.43423	108.4889
**Frg**	100	69.26241	111.4022	132.6036
**Hck**	100	100.0667	132.3898	162.4953
**FAK**	100	96.46324	132.2894	126.2003
**STAT2**	100	126.9072	97.20593	120.5616
**STAT3**	100	109.6658	101.5223	247.599
**STAT 5a**	100	115.5917	122.0649	135.4271
**STAT5 b**	100	93.40943	90.0274	117.703
**STAT5 A/B**	100	121.4369	116.3074	154.2314
**STAT7**	100	110.9859	93.45342	112.7044
**p53 S392**	100	83.20326	356.6267	198.7431
**p53 S 46**	100	105.0083	189.0473	147.1997
**p53 S15**	100	38.12322	190.13	152.7404
**Akt 308**	100	131.3407	172.3985	161.1092
**p70 S6 T421/S424**	100	134.2128	104.9497	134.3285
**p70S6 T229**	100	93.99121	142.352	136.8473
**cJun**	100	107.2125	141.3437	160.8311
**PLC-γ1**	100	263.5613	266.5383	363.6785
**Pyk -2**	100	126.6551	115.0751	152.8664
**p27 T198**	100	0.000239	459.7914	326.2103
**p27 T157**	100	107.9484	480.6506	363.2138
**paxillin**	100	159.0782	250.3517	152.2328
**RSK1/2/3**	100	87.01548	99.49556	89.35632
**RSK1/2 S221 S227**	100	78.24321	111.3611	104.9323
**STAT1**	100	123.3307	100.8336	170.8514
**STAT4**	100	85.94709	101.5377	160.658
**eNOS S1177**	100	156.1881	173.3605	94.30464
**p70 S6 kinase T389**	100	100	74681710	3.26E+08

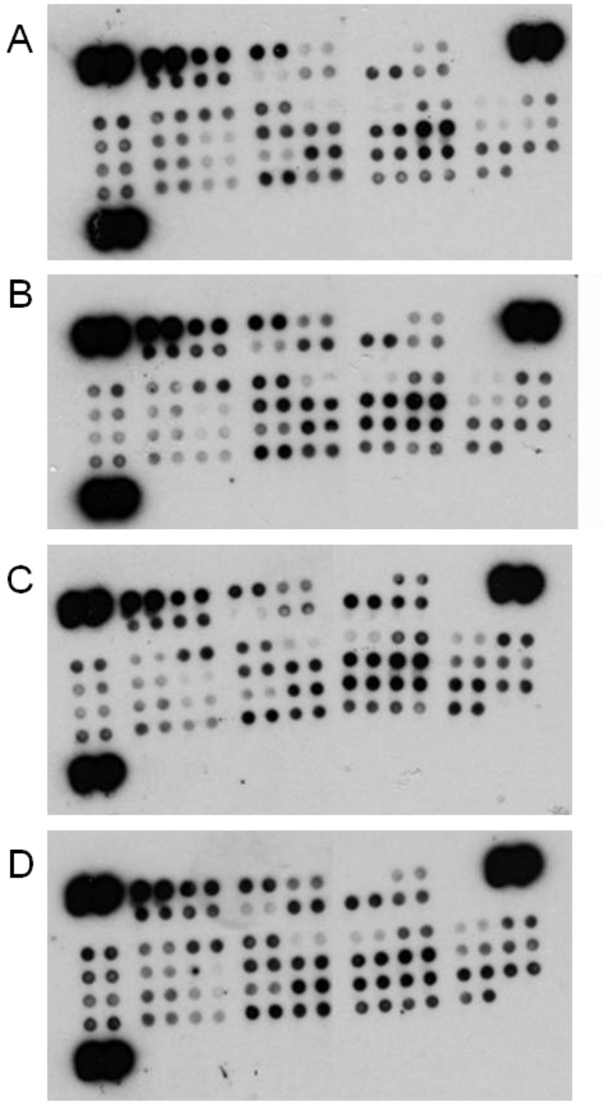

Figure S2: Phospho-kinase array immunoplots. A) VC, B) $AgNW_3$, C)$AgNW_5$, D) $AgNW_{14}$.
